# Effect of Soil Washing Solutions on Simultaneous Removal of Heavy Metals and Arsenic from Contaminated Soil

**DOI:** 10.3390/ijerph17093133

**Published:** 2020-04-30

**Authors:** Kanghee Cho, Eunji Myung, Hyunsoo Kim, Cheonyoung Park, Nagchoul Choi, Cheol Park

**Affiliations:** 1 Research Institute of Agriculture and Life Sciences, Seoul National University, Seoul 08826, Korea; kanghee1226@hanmail.net; 2 Department of Energy and Resource Engineering, Chosun University, Gwangju 61452, Korea; ej6865@naver.com (E.M.); star8538@naver.com (H.K.); cybpark@chosun.ac.kr (C.P.); 3 Construction Technology Research Center, Korea Conformity Laboratories, Seoul 08503, Korea

**Keywords:** mixture solution, sulfuric acid, phosphoric acid, simultaneous removal, heavy metals

## Abstract

In this study, we investigated the feasibility of using a solution of sulfuric acid and phosphoric acid as an extraction method for soil-washing to remove Cu, Pb, Zn, and As from contaminated soil. We treated various soil particles, including seven fraction sizes, using sulfuric acid. In addition, to improve Cu, Pb, Zn, and As removal efficiencies, washing agents were compared through batch experiments. The results showed that each agent behaved differently when reacting with heavy metals (Cu, Pb, and Zn) and As. Sulfuric acid was more effective in extracting heavy metals than in extracting As. However, phosphoric acid was not effective in extracting heavy metals. Compared with each inorganic acid, As removal from soil by washing agents increased in the order of sulfuric acid (35.81%) < phosphoric acid (62.96%). Therefore, an enhanced mixture solution using sulfuric acid and phosphoric acid to simultaneously remove heavy metals and As from contaminated soils was investigated. Sulfuric acid at 0.6 M was adopted to combine with 0.6 M phosphoric acid to obtain the mixture solution (1:1) that was used to determine the effect for the simultaneous removal of both heavy metals and As from the contaminated soil. The removal efficiencies of As, Cu, Pb, and Zn were 70.5%, 79.6%, 80.1%, and 71.2%, respectively. The combination of sulfuric acid with phosphoric acid increased the overall As and heavy metal extraction efficiencies from the contaminated soil samples. With the combined effect of dissolving oxides and ion exchange under combined washings, the removal efficiencies of heavy metals and As were higher than those of single washings.

## 1. Introduction

**Figure 1:**
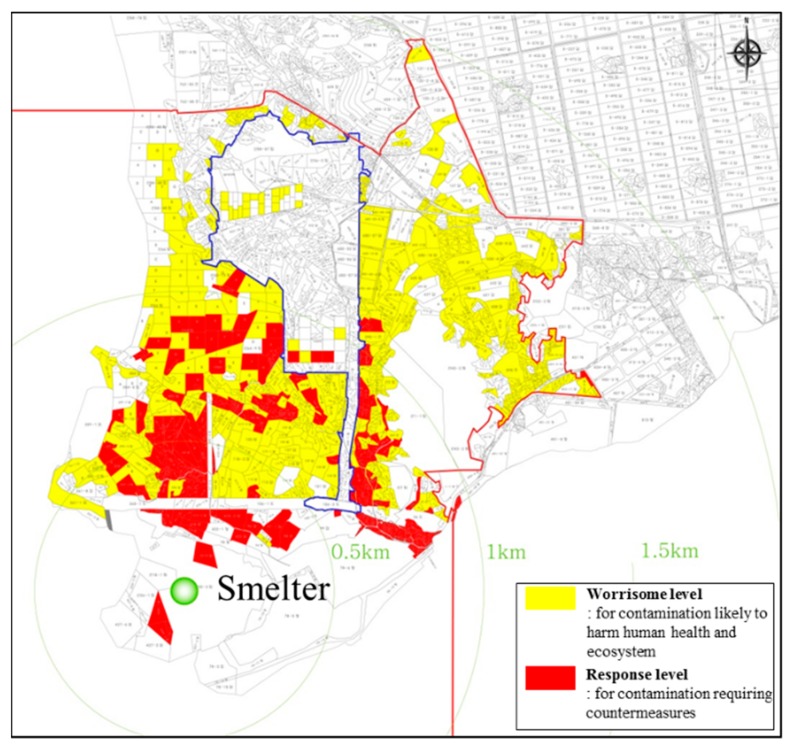
The distribution of heavy metals in soils near a smelter.

The heavy metal contamination of soils is a worldwide problem. Soil contaminated with heavy metals occurred at a smelter site in Korea as a result of smelting for industrialization, which eventually caused long-term effects regarding the pollution of metals in agricultural areas, posing a potential risk to human health [1]. Smelting activities have been responsible for the persistence of extensive soil contamination in modern times. Smelter processes introduce heavy metal contaminants into the environment in the form of gaseous emissions. Contaminants associated with particulates emitted from smelter operations are concentrated in the ultrafine particle fraction, which may be emitted to greater distances into the environment [2]. Soil contamination also occurred at smelter sites, owing to the heavy metals contained in scattered ores, refined residue, and chimney dust. The spatial distribution of contaminants in soils near metal smelters were revealed within approximately 2 km of a smelter in Korea (Figure 1), and the sources of heavy metal contamination were found to be atmospheric dust emissions derived from the smelter, ore dump, and sludge generated from the smelting process [1,2].

The distribution of metal contaminants in the soils surrounding a smelter depends on wind direction and/or velocities, size of particles, and physicochemical characteristics of soils owing to atmospheric deposition [3]. In particular, the highest concentrations of contaminants in soils near smelters occur in the uppermost horizons [4]. Unfortunately, contaminated soils, owing to their varied characteristics, have caused substantial challenges in soil remediation efforts [5]. Therefore, the soil in the contaminated sites must be treated by appropriate remediation techniques to prevent the problems caused by its contaminants. Several recent studies have been conducted using various physical, chemical, and biological soil remediation techniques for heavy-metal-contaminated sites. A variety of remediation techniques have been developed for soil cleanup. The removal of heavy metals from contaminated soil can be performed employing a combination of physical separation and soil washing. The concentration of heavy metals is affected by the particle size of the contaminated soil [6]. Clay-sized fractions can adsorb more contaminants than sand-sized fractions because of their larger specific surface areas. In general, elemental adsorption increases with decreasing particle size. Physical separation, which can reduce the contaminated soil volumes, is cost-effective.

Soil washing involves the use of chemicals to extract contaminated soil. There are various washing agents. Generally, inorganic acids (e.g., sulfuric acid) are used as washing agents for the remediation of heavy metal from contaminated soil, and a strong acidic condition is used to secure ion exchange and the dissolution of soil mineral components. However, acid washing is not effective for As because negatively charged As can be re-adsorbed to the positively charged soil under acidic conditions [7,8]. Thus, As removal using alkaline solutions has been proposed [8], in which As could be directly ion-exchanged with hydroxyl ions. Organic acids (e.g., oxalic acid, citric acid, and ascorbic acid) and chelating agents (e.g., EDTA) have been used for the removal of As-contaminated soils. Among these washing agents, phosphate-containing solutions can be used to extract As from soils. This is because the crystal structures of both As and phosphorus, AsO_4_^3−^ and PO_4_^3−^, are similar, both have tetrahedral form, and both exist in the form of anions in the soil and have similar chemical behavior [9]. There is isomorphic exchange between AsO_4_^3−^ and PO_4_^3−^, which leads to adsorption competition [10]. Previous studies on soil washing of As-contaminated soils with oxalic acid [7], EDTA [11], NaOH [12], and phosphate solutions [9] have focused on the effects of various experimental parameters and removal mechanisms by respective washing agents on As removal efficiency. Only a few have investigated the simultaneous removal of both anionic As and cationic heavy metals from contaminated soils.

Although strong acids can effectively leach heavy metals from soil, this process will lead to changes in the physical and chemical properties of the soil and may cause permanent damage. Soil washing could have detrimental effects on soil functions by decreasing soil enzyme activities and micronutrients [10]. Nonetheless, the remediation of heavy metals in contaminated soil is not only necessary to control contaminants but also enhances the stabilization of the contaminants within the bulk matrix by breaking pollutant linkages. In particular, soil contaminated with smelter-derived particulates are chemically and mineralogically complex materials [13]. Lee et al. (2019) reported that contaminated soil contained sulfide minerals, including Fe-(oxy) hydroxides and rutil (TiO_2_), with small amounts of pyrite (FeS_2_), ilmenite (FeTiO_3_), sphalerite (ZnS), and chalcopyrite (CuFeS_2_) [1]. In addition, soil contamination was caused by scattering ores, refining residues, and chimney dust containing arsenic trioxide (As_2_O_3_). In this study, we took samples for contaminated soil that contained As, Cu, and Pb levels higher than the “Korean worrisome level” presented by the Korean Soil Environment Conservation Act. Therefore, the development of effective washing reagents that can simultaneously remove heavy metals and As is required. The objective of this study was to investigate the simultaneous extraction of both anionic As and cationic heavy metals from contaminated soils. In detail, the simultaneous extraction of As, Cu, Pb, and Zn from contaminated soils was tested with the most commonly used sulfuric acid and phosphoric acid, combined with inorganic acids.

## 2. Materials and Methods

### 2.1. Soil Sampling and Characterization

The contaminated soil was sampled from a smelter located in Janghang, Korea, where heavy metal contamination is a major environmental problem [1]. Soil contamination represents a direct sink for contaminants emitted to the atmosphere by smelters. In addition, soil contamination was caused by scattered ores, refined residues, and chimney dust containing heavy metals (Figure 1). During smelting, heavy metals, such as Cu, Pb, Ni, and Zn, are typically released into the surrounding environment [2]. For this reason, the contaminated soil (10 kg) was collected from the surface layer (0–30 cm). A representative contaminated sample at a given point was obtained by mixing 10 soil samples collected within a circle of approximately 10 m in diameter, centered at that point. The collected soil was mixed and homogenized, air-dried at room temperature, and then sieved through a 2 mm screen.

The particle size analysis of the contaminated soil revealed the composition of sand (37.60%), silt (43.72%), and clay (21.68%), which represents the textural classification of loam soil. The primary physical and chemical properties of the soil were as follows: pH 6.40, organic matter 2.74%, and cation exchange capacity (CEC) 19.65 cmol/kg (Table 1).

The characteristics of soil contaminated with heavy metals are summarized in Table 2. The heavy metal (Cu, 252.40 ± 2.55 mg/kg; Pb, 490.74 ± 3.55 mg/kg) and As (139.53 ± 2.65 mg/kg) concentrations exceeded the cleanup levels (25 mg/kg for As, 150 mg/kg for Cu, and 200 mg/kg for Pb) of the Korean Soil Environment Conservation Act.

The mobility of metals is affected by the particle size of the soil. Silt or clay soil can adsorb more contaminants than sand because of their larger specific surface area; therefore, the concentrations of contaminants are high. In addition, heavy metals of the smallest size fraction (<0.053 mm) are difficult to remove owing to their strong adsorption [15]. The size separation, a factor that affects enhanced soil washing, can concentrate contaminants into smaller volumes. If the smallest size fractions are properly separated, the overall effectiveness of soil washing is enhanced. Oversized material (stones) was separated from the soil by sieving through a 4 mm screen. For the separation procedure, the air-dried sample (300 g) was weighed and dry-sieved. Three hundred grams of soils were placed on the top of a nest of sieves and fractionated into six aggregate sizes by vibrating screen instrument. We evaluated the removal efficiency of contaminants in the soil fractions, and the soil particles were divided into seven fraction sizes, <2.0, 2–1, 1–0.5, 0.5–0.15, 0.15–0.106, 0.106–0.053, and <0.053 mm.

**Figure 2:**
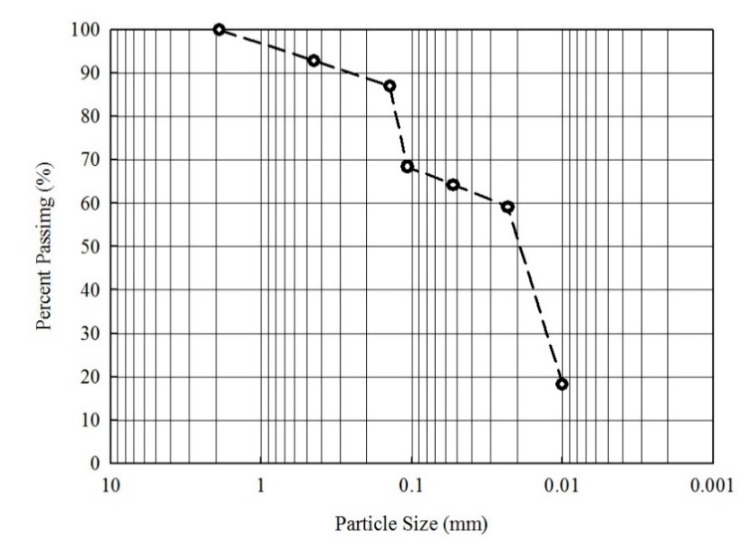
Particle-size distribution of contaminated soil.

The distribution of the soil was determined by the weight of each size after sieving, corresponding to the fractions of 7.17%, 5.87%, 18.57%, 4.16%, 5.08%, 40.88%, and 18.27% of the soil sample, respectively. The distribution of weight according to the seven particle sizes is given in Figure 2. Overall, the distribution characteristics were significantly related to the particle size and contaminated-soil concentration. The two particle size fractions in the ±0.106 mm range exhibited differences in As, Cu, Ni, Pb, and Zn concentrations. This indicated that heavy metals were more likely to be enriched in the smaller particle-size fractions.

### 2.2. Batch Experiment: Effect of Soil Characteristics and Washing Solution

A batch experiment was conducted to observe the release of heavy metals (Cu, Pb, and Zn) and As from the contaminated soil. The washing agents—sulfuric acid, phosphoric acid, and two acid mixtures consisting of sulfuric acid and phosphoric acid—were used as washing solutions. This selection was based on the consideration that they were typical and widely used washing agents [16]. 

A batch experiment for the remediation of heavy metals and As from the contaminated soils were investigated in terms of acid solution and particle-size fraction. Experimental conditions were determined based on previous results from soil washing [7,8]. A range of sulfuric acid concentrations (0.1–0.6 M) and washing periods (~120 min) were used to determine the optimum washing conditions for heavy metal and As removal. In addition, water washing was used as a control under the same conditions. Soil-washing experiments were performed with 5 g of soil with 35 mL of washing solution in a 50 mL conical tube. The suspensions were then shaken at 250 rpm for 2 h at 20 °C in a shaking incubator. The suspensions were centrifuged at 3000 rpm for 10 min, and the supernatants were filtered through a syringe filter. Concentrations of heavy metals and As in the effluent were determined using atomic absorption spectrophotometry (AAS). Each experiment was performed in duplicate and repeated at least twice. The relative standard deviation (*n* = 2) of duplicates was less than 10%.

The amount of metal ion extracted was calculated via the following equation:

(1) $$L=\frac{M_{1}}{M_{0}}\times 100$$

where L is the extraction percentage of metal ion and *M*_0_ and *M*_1_ correspond to the metal ion content of the sample before and after leaching.

The rate of metal effluent was determined by the following equation,

(2) $$E=E_{I}\left(1-e^{-kt}\right),$$

where E (%) is the metal ion concentration in the extraction solution at time *t*, *E_I_* (%) is the maximum concentration of metal ion, and *k* (min^−1^) is the extraction rate constant.

### 2.3. Analysis Method

The soil pH was analyzed by mixing with deionized water at a ratio of 1:5 (soil:deionized water). EPA method 9081 was used to analyze the CEC of the soil. Soil particle analysis was performed by sieving and weighing the soil (300 g) with seven fractions (<2.0, 2–1, 1–0.5, 0.5–0.15, 0.15–0.106, 0.106–0.053, and <0.053 mm). The fractionation of the metals in soil was determined according to the traditional sequential extraction (Tessier’s method) procedure of Standards and Measures [17], which divides the heavy-metal-bound soil fractions into five categories (exchangeable fraction, bound-to-carbonate fraction, bound-to-Fe-Mn-oxide fraction, bound-to-organic-matter fraction, and residual fraction). After the washing experiments, the heavy metals and As in the soil were measured based on the Korean standard test methods (aqua regia) and compared with the Korean warning standards for forest land and residential areas [18]. The total concentrations of selected heavy metals (Cu, Pb, and Zn) and As in 1 g soil samples were extracted using HCl and HNO_3_ at 3:1 ratio (i.e., aqua regia), and the extracts were filtered for analysis. The solution was analyzed through AAS (AA-7000, Shimadzu, Japan). Total arsenic analysis was performed using graphite furnace atomic absorption spectrometry (GFA-7000A, Shimadzu, Japan) at λ 193.7 nm.

## 3. Results and Discussion

### 3.1. Characteristics of Soil: Effect of Particle-Size Fraction

**Figure 3:**
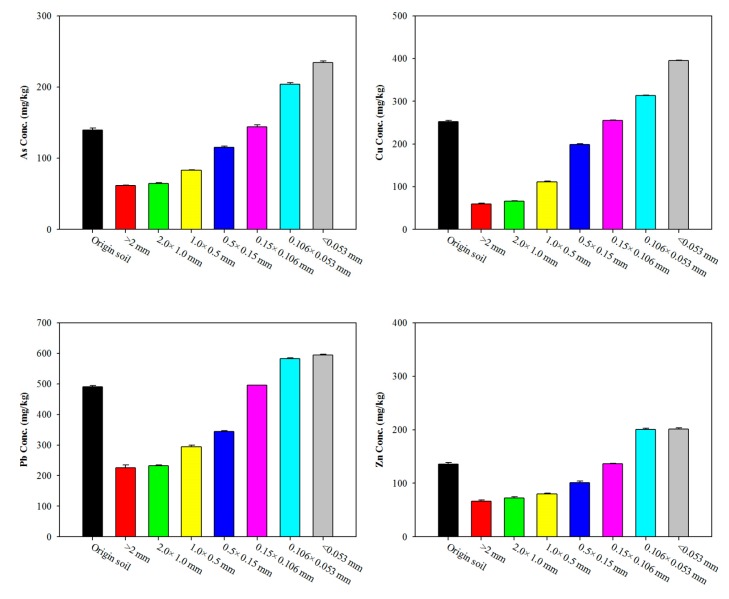
Concentration distribution of As, Cu, Pb, and Zn according to particle size.

The soil particles were categorized into seven fraction sizes: <2.0, 2–1, 1–0.5, 0.5–0.15, 0.15–0.106, 0.106–0.053, and <0.053 mm. As shown in Figure 2, the 0.106–0.053 mm particle was the dominant fraction size at 40.88%. This particle size fraction contributed the largest mass loadings, with the loadings in the order of >0.106 mm (40.85%) and <0.053 mm (18.27%). The concentrations of heavy metals and As in the soil increase with decreasing particle size, with the highest concentration observed in the <0.053 mm fraction (Figure 3).

**Figure 4:**
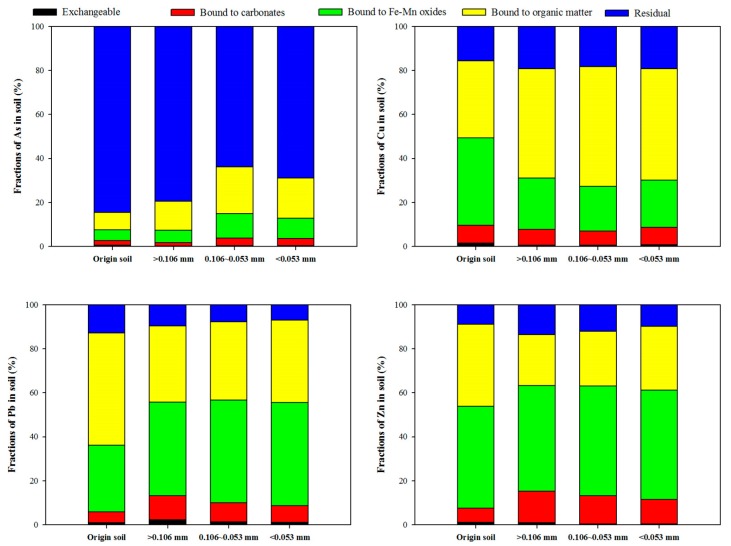
Concentration distribution of copper, lead, and As according to particle size.

Similar trends for the concentration of heavy metals and As for the particle size fraction were found compared with the original soil and <0.106 mm in the soil. Concentrations of As and Pb for all particle size fraction soils exceeded the cleanup level of the Korean Soil Environment Conservation Act. The concentration of Cu in the < 0.15 mm fraction was also exceeded. The percentage of heavy metals and As in the five sample fractions is shown in Figure 4. The Cu, Pb, and Zn in the soil were primarily associated with Fe-Mn oxides (Cu 39.84%; Pb 30.44%; and Zn 46.23%, Step 3) and organic matte (Cu 34.84%; Pb 51.01%; and Zn 37.31%, Step 4), whereas the As in the soil was primarily associated with the residual (84.63%, Step 5). In particular, the largest mass loadings of particle fractions were divided into >0.106 mm, 0.106–0.053 mm, and <0.053 mm. Similar trends for the concentrations of Cu, Pb, and Zn for the particle size fraction were observed, whereas As showed a slow increase in the organic matte bound (Step 4) with decreased particle size fraction.

**Figure 5:**
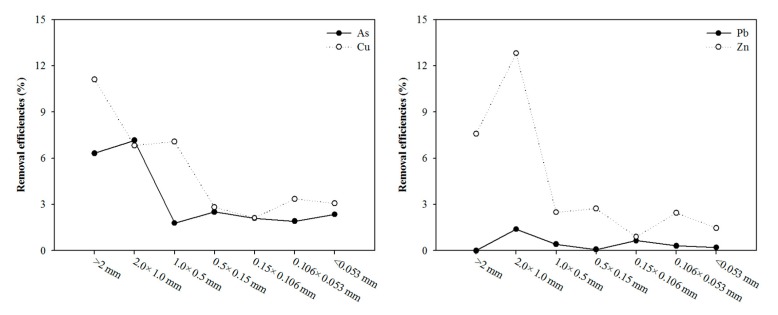
Effect of the size fraction on the extraction of As, Cu, Pb, and Zn from the batch test with water.

For water extraction and the <1.00 mm fraction (Figure 5), removal efficiencies generally reached 10%, which is attributed to the low concentration of the exchangeable bound fractions of Zn, Cu, and As in soil. However, Pb removal with water was small. In contrast, the metal removal efficiencies in the >1.00 mm fraction were less than 3%. This phenomenon may be related to the larger surface area of the >1.00 mm fraction and the oxides produced by Si–OH and Al–OH, which can adsorb more metals in soils [19]. Inorganic acids could extract metals by the dissolution of metal ions and direct ion exchange. Some studies have shown that clay minerals [7], organic matter [10], and Fe–Mn oxides [20] usually act as adsorption sites, implying a robust adsorption mechanism with the toxic elements. Liao et al. (2016) showed that soil washing is suitable for porous sandy soils with high permeability. Once the soil has a high content of organic matter or clays (>30%), soil washing is considered ineffective for heavy metal remediation [6]. However, the soil particles finer than 0.02 mm can be washed but only if the contaminants can be solubilized in suitable reagents.

### 3.2. Effect of Washing Solution: Sulfuric Acid, Phosphoric Acid, and Mixing Washing Solution

**Figure 6:**
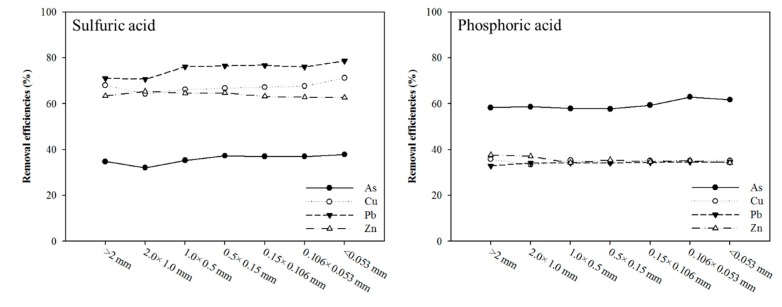
Effect of size fraction on the extraction of As, Cu, Pb, and Zn from the batch test with 0.6 M sulfuric acid and 0.6 M phosphoric acid.

Preliminary extraction experiments were conducted. Phosphoric acid (concentration, 0.6 M; reaction duration, 120 min; 250 rpm; and various soil particle size fractions) was applied to compare with the sulfuric acid washing solution. The removal efficiencies of the different particle sizes were presented in the decreasing or similarity order of As > Cu ≈ Pb ≈ Zn. There was a distinct difference among the heavy metal and As removal efficiencies, whereas sulfuric acid (concentration, 0.6 M; reaction duration, 120 min; 250 rpm; and various soil particle size fractions) exhibited removal efficiencies of the different particle sizes in the decreasing order of Pb > Cu > Zn > As (Figure 6).

**Figure A1:**
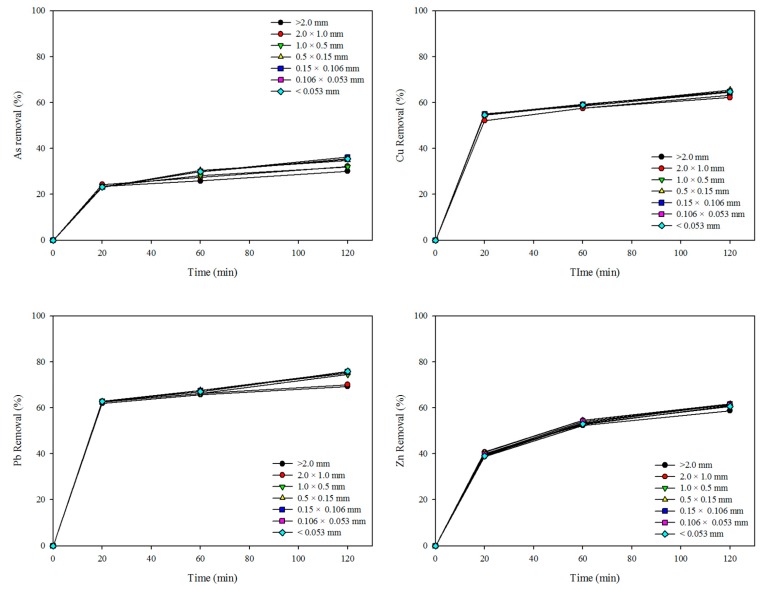
Effect of the size fraction on the extraction of As, Cu, Pb, and Zn from the batch test with 0.6 M sulfuric acid.

Washing time influenced the removal of heavy metals in contaminated soil. The removal efficiencies of heavy metals increased with reaction time up to 120 min, which can be attributed to the extraction of more heavy metals from the soil with each particle size. Based on the experimental results, the parameters of the extraction experiment conditions were estimated using Equation (2). As shown in Table A1, the extraction of each particle size has a high correlation coefficient (R^2^). The extraction rate constant by sulfuric acid from each particle size was in the order of Pb > Cu > Zn > As. In addition, the removal of heavy metal with decreased particle size shows the slow rate (Figure A1).

**Figure 7:**
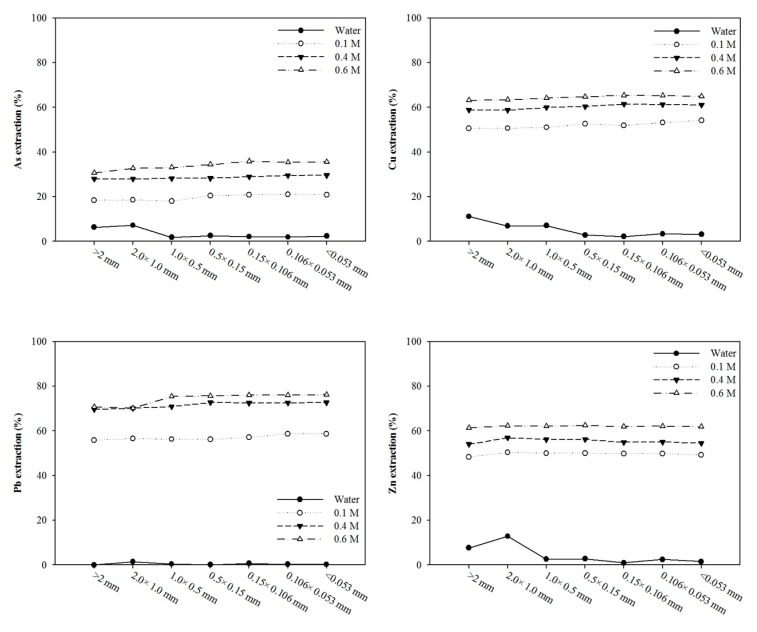
Effect of sulfuric acid concentrations on the extraction efficiencies of As, Cu, Pb, and Zn from the contaminated soil. The soil-washing conditions were as follows: *S*/*L* = 1:7, stirring speed: 250 rpm, and washing time: 120 min.

The particle size of soil fractions has significant effects on heavy metal removal from contaminated soils. However, it can be noticed in Figure 7 that the metal removal in the soil does not change depending on particle size for sulfuric acid or phosphoric acid. 

**Figure 8:**
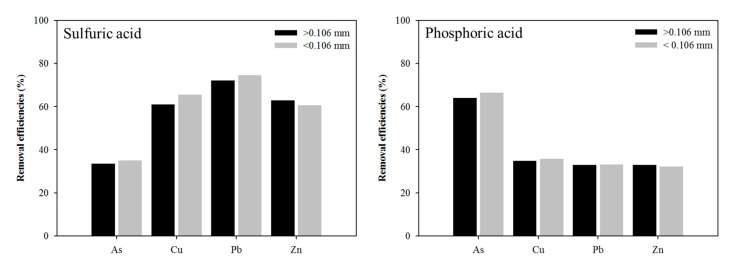
Effect of the size fraction (±0.106 mm) on the extraction of As, Cu, Pb, and Zn from the batch test with 0.6 M sulfuric acid and 0.6 M phosphoric acid.

Generally, a high washing solution concentration would induce a high removal efficiency. The removal efficiencies of heavy metals increased with sulfuric-acid concentration, which can be attributed to the extraction of more heavy metals from the soil. The metal removal efficiencies slowly increased with increasing concentration and reached a maximum level at 0.6 M (35.81% for As, 65.35% for Cu, 76.19% for Pb, and 62.38% for Zn). High removal efficiency was achieved by sulfuric acid. However, sulfuric acid was ineffective for As removal. Compared with each inorganic acid, As removal from soil by washing agents increased in the order of sulfuric acid (35.81%) < phosphoric acid (62.96%). In addition, extraction efficiencies of ±0.106 mm fraction were investigated for inorganic acid through the consideration of mass loading by particle size fraction. Similar trends for the removal efficiencies of particle size fraction were also found compared with those of sulfuric acid and phosphoric acid in the experiments (Figure 8). 

This indicated that As in the soil could be extracted by enhancing the removal efficiencies of As because of the similar chemical properties between As and phosphorus [8]. This enhancement may be related to these phosphates in inorganic acid. The removal of As during the soil washing in the presence of sulfuric acid occurs such that oxyanionic As can be re-adsorbed into the soil under acidic conditions [9]. However, phosphoric acid may be attributed to a phosphate that can compete with As for sorption, which could be extracted by enhancing the removal efficiency of As. Some studies [21,22] have shown that the simultaneous extraction of As and heavy metals (Cu, Fe, Pb and Zn) is enhanced by reducing agents (oxalate, ascorbic acid, and dithionite) and chelating agents (EDTA). Kim et al. (2016) showed that the combination of dithionite and EDTA can effectively extract As and heavy metals simultaneously from soils under a wide range of pH conditions.

**Figure 9:**
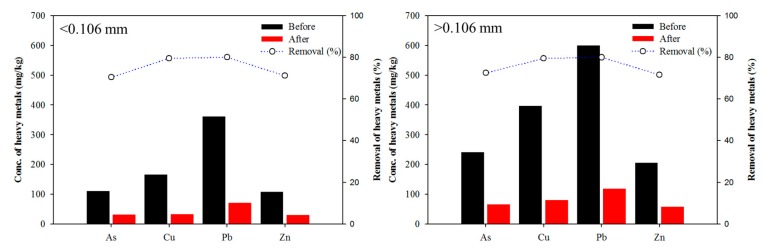
Effect of size fraction (±0.106 mm) on the extraction of As, Cu, Pb, and Zn from the batch test with mixture solution (0.6 M sulfuric acid and 0.6 M phosphoric acid, 1:1).

Sulfuric acid at 0.6 M was adopted to combine with 0.6 M phosphoric acid to obtain the mixture solution (1:1) that was used to determine the effect for the simultaneous removal of both heavy metals and As from the contaminated soil. The removal efficiencies of As, Cu, Pb, and Zn were 70.5%, 79.6%, 80.1%, and 71.2%, respectively. In addition, similar trends for the removal efficiencies for particle size fraction (±0.106 mm) were found (Figure 9).

The combination of sulfuric acid with phosphoric acid increased the overall As and heavy metal extraction efficiencies from the soil samples. The main reason is the increased mobility of the metals under the acidic conditions. With the increased proportion of inorganic acid, the concentration of H^+^ provided by phosphoric acid increases, as does the acid solubility; this can effectively promote the dissolution of heavy metals from soil. The addition of phosphoric acid to sulfuric acid can increase the extraction of As and heavy metals from the soil samples by enhancing the dissolution of the oxides, which strongly bind to As and heavy metals in the soils. The washing solution that included the competitive oxyanions was favorable for the removal of oxyanionic As and cationic heavy metals. The removal of As during the soil-washing process in the presence of sulfuric acid and phosphoric acid occurs through a series of reactions that can be considered. Oxyanionic As could be re-adsorbed into the soil under sulfuric acid conditions. However, competitive oxyanions, such as phosphate and sulfate from mixture solution, enhanced As extraction. Heavy metals and As are incorporated into the strong bonds with mineral surfaces. Therefore, it may be broken effectively by the inorganic acid. In addition, size separation removed the > 0.106 mm fraction including the contaminants, which significantly enhanced the removal efficiencies from the volume of contaminated soil.

## 4. Conclusions

In this study, sulfuric acid and phosphoric acid were investigated to remove heavy metals and As from contaminated soil. Inorganic acid was the applied washing solution since the heavy metals and As in the soil were strongly associated with the high proportions of the Step 3 and Step 4 fractions for heavy metals and the Step 5 fraction for As through the sequential extraction procedure. Sulfuric acid was more effective in extracting heavy metals (Cu, Pb and Zn) than in extracting As. However, phosphoric acid was not effective in extracting heavy metals, whereas phosphoric acid achieved higher removal efficiencies of As under the single washing solution, owing to its dissolving capacity of soil minerals for the similar chemical properties between As and phosphorus. The competitive phosphate replacement of oxyanion As in soil exchange sites resulted in the extraction of oxyanions and high As removal. Therefore, the enhanced mixture solution metal extraction from contaminated soils by sulfuric acid and phosphoric acid were investigated to effectively and simultaneously remove both heavy metals and As from the contaminated soil. The addition of phosphoric acid greatly enhanced the extraction of heavy metals and As from the soils, owing to the increased mobility of the metals through the enhanced dissolution of the soil oxides under acidic conditions. Because of the combined effect of dissolving oxides and ion exchange under combined washing, the removal efficiencies of heavy metals and As were higher compared with that of single washings.

Soil washing approaches are usually based on site-specific study. Before designing a full-scale washing plant, a feasibility analysis is required. At this stage, several laboratory and analytical determinations are performed to define the properties of both the pollutant and the soil. For the simultaneous removal of As and heavy metals, an optimal remedial condition should be set up. As mentioned above, this paper describes a feasibility study of washing techniques in a soil affected by speciation of heavy metals in different soil particle-size fractions. The soil pollution in the study site is relevant from the point of view of the volume of soil affected. In addition, the combination of sulfuric acid with phosphoric acid increased the overall As, Cu, Pb, and Zn extraction efficiencies from the contaminated soil. 

## Figures and Tables

**Table 1:** Physical and chemical properties of soil sample.

pH	Organic Matter (%)	CEC (cmol/kg)	Sand (%)	Silt (%)	Clay (%)
6.40	2.74	19.65	37.60	43.72	21.68

**Table 2:** Distribution of heavy metal concentrations according to particle size fraction, concentration of original soil, and Korean threshold values.

Elements	As (mg/kg)	Cu (mg/kg)	Ni (mg/kg)	Pb (mg/kg)	Zn (mg/kg)
Korean limit ^(1)^	25	150	100	200	300
Original soil	139.53 ± 2.65	252.40 ± 2.55	17.86 ± 0.62	490.74 ± 3.55	135.37 ± 3.58
+0.106 mm	112.10	167.64	14.24	362.50	109.46
−0.106 mm	241.94	394.36	34.75	600.61	207.09

^(1)^ “Korean worrisome level” of the Korean Soil Environment Conservation Act [14].

## Appendix A

**Table A1:** Summary of extraction parameters for As, Cu, Pb, and Zn using Equation (2) with sulfuric acid.

Elements.	Size Particle Fraction	E_I_ (%)	K (min^−1^)	R^2^
As	>2 mm	30.14	0.0873	0.9840
	2.0 × 1.0 mm	32.20	0.0801	0.9830
	1.0 × 0.5 mm	31.96	0.0723	0.9908
	0.5 × 0.15 mm	34.79	0.0558	0.9923
	0.15 × 0.106 mm	36.19	0.0534	0.9813
	0.106 × 0.053 mm	35.31	0.0550	0.9864
	<0.053 mm	35.44	0.0548	0.9861
Cu	>2 mm	63.29	0.0977	0.9938
	2.0 × 1.0 mm	62.22	0.1011	0.9958
	1.0 × 0.5 mm	64.55	0.1120	0.9935
	0.5 × 0.15 mm	65.54	0.1027	0.9921
	0.15 × 0.106 mm	64.88	0.1048	0.9939
	0.106 × 0.053 mm	64.72	0.1076	0.9945
	<0.053 mm	64.73	0.1056	0.9943
Pb	>2 mm	69.33	0.1240	0.9980
	2.0 × 1.0 mm	70.10	0.1238	0.9977
	1.0 × 0.5 mm	74.43	0.1081	0.9906
	0.5 × 0.15 mm	75.01	0.1032	0.9927
	0.15×0.106 mm	75.74	0.1025	0.9910
	0.106 × 0.053 mm	75.59	0.1034	0.9900
	<0.053 mm	75.85	0.1036	0.9895
Zn	>2 mm	58.75	0.0550	0.9951
	2.0 × 1.0 mm	61.42	0.0557	0.9943
	1.0 × 0.5 mm	60.96	0.0549	0.9906
	0.5 × 0.15 mm	61.73	0.0548	0.9938
	0.15 × 0.106 mm	61.56	0.0521	0.9922
	0.106 × 0.053 mm	61.29	0.0534	0.9940
	<0.053 mm	60.64	0.0523	0.9932
